# Is the Family Size of Parents and Children Still Related? Revisiting the Cross-Generational Relationship Over the Last Century

**DOI:** 10.1007/s13524-019-00767-5

**Published:** 2019-03-13

**Authors:** Eva Beaujouan, Anne Solaz

**Affiliations:** 1 Vienna University of Economics and Business (WU), Department of Socioeconomics/Wittgenstein Centre for Demography and Global Human Capital, Building D4, 3rd Floor, Welthandelsplatz 1, 1020 Vienna, Austria; 2 Institut National d’études Démographiques (INED), 133, boulevard Davout, F-75020 Paris, France

**Keywords:** Intergenerational transmission, Fertility, Family size, Parents, Children, Gender

## Abstract

In most developed countries, the fertility levels of parents and children are positively correlated. This article analyzes the strength of the intergenerational transmission of family size over the last century, including a focus on this reproduction in large and small families. Using the large-scale French Family Survey (2011), we show a weak but significant correlation of approximately 0.12–0.15, which is comparable with levels in other Western countries. It is stronger for women than men, with a gender convergence across cohorts. A decrease in intergenerational transmission is observed across birth cohorts regardless of whether socioeconomic factors are controlled, supporting the idea that the family of origin has lost implicit and explicit influence on fertility choices. As parents were adopting the two-child family norm, the number of siblings lost its importance for having two children, but it continues to explain lower parity and, above all, three-child families. This suggests that the third child has increasingly become an “extra child” (beyond the norm) favored by people from large families.

**Electronic supplementary material:**

The online version of this article (10.1007/s13524-019-00767-5) contains supplementary material, which is available to authorized users.

## Introduction

One salient feature of fertility in post-transition societies is that people from large families tend to have more children, and those from small families have fewer (Murphy 2013). Although the intergenerational links between a mother’s and her children’s fertility have been reported as negligible in most natural-fertility populations, a positive intergenerational fertility correlation emerged during the demographic transition (Murphy 1999; Rotering 2017). This emergence has been attributed to the shift from social to individual control of family behavior in more permissive societies (Jennings et al. 2012; Kohler et al. 1999; Udry 1996; Van Bavel and Kok 2009). The increased ability to choose fertility outcomes, although still limited, created the potential for a transmission of behaviors from parents to children. A positive and generally significant correlation between parents’ and children’s fertility levels, ranging from .10 to .20, has been observed in several countries with very different fertility contexts since the late nineteenth century (review in Murphy 2013). In France, the first studies of family size transmission, which date back to the 1980s, found that family size increased with the number of brothers and sisters among women born between World Wars I and II (Desplanques 1985; Deville 1979).

The positive link between the number of siblings and one’s own fertility seems to have withstood the widespread changes that occurred in families in developed countries during the twentieth century (Dahlberg 2013; Murphy 2013). Nevertheless, the diffusion of the two-child model among 1930s to 1950s birth cohorts and the individual freedom in family formation that emerged during the second demographic transition may have modified the extent to which family behavior is inherited from parents. The dynamics of the intergenerational relationship over the century thus deserve fuller investigation.

In this article, we explore the strength of the relationship between parents’ and children’s family size over the twentieth century in France. We take advantage of the huge sample size of a French retrospective survey, which was a supplement to the 2011 census, covering almost one century of French fertility. Our long-term approach links the fertility of French birth cohorts born since 1922 and that of their parents who were born from the end of the nineteenth century onward. Several components, whether genetic or related to socialization during childhood, are reported to explain the fertility transmission from parents to children. We provide a nuanced analysis of the trends in the intergenerational transmission of fertility, taking into account both women and men. Modeling the effect along the fertility distribution, we analyze which family sizes are more sensitive to the origin family size across birth cohorts. We systematically test the sensitivity of intergenerational family size to the introduction of controls for the parents’ and children’s socioeconomic characteristics.

## Previous Findings About the Parent-Child Fertility Relationship Over Time

Research has provided mixed evidence about the change in intergenerational family size correlation over the last century. Murphy’s (1999) meta-analysis showed an increasing trend. Murphy and Wang (2001) confirmed that among women born in the mid-twentieth century, the relationship between the number of siblings and children has tended to become stronger over time in a few developed countries. However, Dahlberg (2013) established that intergenerational continuity in fertility behavior remained stable in Sweden between the 1940 and 1955 birth cohorts for men as well as women. Finally, using a comprehensive collection of data sets, Murphy (2013) showed a stable relationship in the most recent cohorts (until approximately the 1965 birth cohort), suggesting that the previously increasing trend did not last. Most of these studies are limited because their data covered only a few cohorts that have not necessarily reached their final number of children. Our first contribution is to observe the strength of the intergenerational transmission of fertility over time based on a large-scale survey and completed fertility data.

Although most research has focused on the overall correlation, the way family size is reproduced may also vary across parities. For Britain, Booth and Kee (2009) found that an increase in the origin-family size particularly results in an increase in own family size for women with few children (first 10 % of the distribution) and several children (second half of the distribution). Although they did not directly study the intergenerational correlation for family size, Breton and Prioux (2009) also showed that in Europe, the probability of having one child is highest among individuals with few siblings and, particularly, only-children. In addition, the probability of having a third child is higher among those who have many siblings (Breton and Prioux 2002). These studies suggest that the two extremes of the fertility distribution drive the intergenerational relationship. Was this always the case over the last century? Our second contribution is to measure the extent to which the reproduction of parental fertility has differed over time along the fertility distribution, which should give good insight into the underlying mechanisms of fertility transmission.

Most of the literature has focused on women only, but studies including both sexes or the male partner’s characteristics have shown that the number of siblings plays a role for both sexes (Booth and Kee 2009; Jennings and Leslie 2012; Nisén et al. 2013; Rotering 2017). Across the twentieth century, the intergenerational correlation was higher for women than for men. This corresponds to the gender role approach, which suggests that women are generally more involved in the family sphere than men (Goldscheider et al. 2015) and might thus be more likely to reproduce the parental model. However, recent studies have found that the stronger influence of women in couples’ fertility decisions has faded (see, e.g., Bauer and Kneip 2013). This challenges results that indicate a stronger effect of parental family size for women than for men, especially in the recent period and in countries such as France, which ranks among the upper third of countries in Europe for gender equality norms (Matysiak and Węziak-Białowolska 2016).

Many individual characteristics may be involved in the transmission of fertility from parents to children (Anderton et al. 1987). Recent research on exogenous variations in fertility based on twin instrumental variables estimates suggested that the transmission can be partly attributed to mediators or to characteristics that parents and their children share (Cools and Hart 2017; Kolk 2015). Notably, one would expect the correlation in family size to be altered by socioeconomic characteristics of both the respondent and his/her parents, so it is essential to consider these factors (Murphy and Wang 2001). First, parental characteristics can act directly on their children’s own fertility and may also act indirectly as moderators in the relationship between parental family size and child family size (Fritz and Lester 2016). Indeed, the magnitude of the association between parents’ and respondent’s fertility could vary depending on parental socioeconomic specificities. For instance, in France among women born at the beginning of the twentieth century, the transmission of family size used to be less pronounced for the upper social classes (Desplanques 1985; Deville 1979). Second, the respondent’s characteristics directly affect own family size. A few studies have found a negative relationship between number of siblings and educational attainment, although this result is not systematic (Blake 1989; Gary-Bobo et al. 2006). Education may thus be linked to the sibship size and thus may act as mediator in the relationship between parental family size and child family size. Finally, controlling simultaneously for parent’s and child’s characteristics allows us to take into account some of the traits shared at the family level and assumed to be one of the main mechanisms behind the intergenerational transmission of fertility (Kolk 2015).

## Mechanisms of the Change: Research Hypotheses

Different mechanisms may have prevailed in leading the century’s trends of intergenerational correlation in number of children, overall or associated with each family size. In this study, we do not claim to unravel the impacts of the likely components on the strength of the intergenerational fertility transmission over time, but we describe their possible effects. Table 1 summarizes the expectations about each possible mechanism on both overall and parity-specific trends of parent-child transmission of fertility. The direction of the relationship varies with the processes described in this section, and the table will help us discuss the results in light of the possible driving forces at the end of the article.

**Table 1:** Expectations about the change in intergenerational correlation across birth cohorts

	Overall:	Family Sizes		
	Any Family Size	Small Family	Two Children	Large Family
Second Demographic Transition Context				
Delay in childbearing age	–			
Diffusion of two-children norm/selection		+	–	+
Genetic Heritability	+			
Socialization	–	–		–

### Fertility Context Over the Last Century

The fertility context has changed considerably over the last century in France. The completed fertility of women born in the 1900 birth cohort was 2.1 children per woman. It increased gradually to 2.65 in the 1930 birth cohort, followed by a strong decrease to 2.0 children per woman born in 1970 (Daguet 2002; Ediev et al. 2012). A phase marked by an in-depth transformation of family behaviors—including the rise in divorce, the postponement of family formation and spread of unmarried cohabitation, and a rapid increase in childlessness—was initiated in the cohorts born after World War II (Lesthaeghe 2010; Sobotka 2008; van de Kaa 1987). Advances in reproductive control represented an important channel of this second demographic transition. Although research has shown that women born at the beginning of the twentieth century already controlled their family size in France and in most developed countries (Bonvalet et al. 2014; Makay 2015; Van Bavel and Reher 2013), the diffusion of oral contraception since the 1970s—and to a lesser extent, the legalization of abortion in France in 1975—gave couples more options to decide on family timing and size. Moreover, the recent development of assisted reproduction might enable an increasing number of couples who have trouble conceiving to reach their desired number of children (te Velde et al. 2012). These advances have certainly allowed for better planning of pregnancies and may also have reduced the gap between the desired and actual number of children (Bongaarts 2001). With fewer practical constraints, people have more freedom to have an actual number of children close to that of their parents if they so wish. We consider that these improvements in control of reproduction will make it easier for people to reproduce—or not—their parents’ childbearing behavior.

In France, like in other Western countries, one of the key features of the second demographic transition was a delay in family formation (Sobotka 2008), which could also suggest more autonomy from one’s family at the time of entry into union and thus a weakening influence of childhood background. For instance, children’s and parents’ family size dissociate as children grow older (Dahlberg 2013), as do intended family size and parents’ fertility (Lois and Arránz Becker 2013; Régnier-Loilier 2006). With the increasing delay in family formation and first parenthood observed from the 1950s cohorts onward in many countries, people may have become, on average, more detached from parental influence at childbearing ages than some decades ago (Billari and Liefbroer 2010; Prioux 2006) and less dependent on parental and social approval. The decoupling of childbearing and marriage that took place in that period, which was particularly marked in France (Coleman 2013; Sobotka et al. 2015), also predicts a detachment from traditional family behaviors.

*The delay in family formation and the diversification of forms of partnership suggest a weaker correlation with parents’ fertility for cohorts who lived through the second demographic transition than for cohorts born before World War II.*

One notable and widespread feature of European fertility is that the two-child model spread massively from the 1930s birth cohorts to the 1950s birth cohorts (David and Sanderson 1987; Frejka 2008; Van Bavel et al. 2015). In France, although three-child families remained relatively widespread, approximately 4 in 10 women born in the 1950s and later had two children (Masson 2013). The transmission mechanisms of family size can be affected by this massive change: Boehnke et al. (2007) argued that intergenerational transmission effects are stronger for families who differ from the normative *Zeitgeist*. The effects within two-child families should become weaker relative to other family sizes because many people follow the normative two-child family model regardless of their origin family size.

*The diffusion of the normative family size model and the resulting selectivity of other family sizes would thus imply that correlations within small (no-child and one-child) and large families should become stronger across cohorts while being weaker for two-child families*.

### Genetic Heritability

Genetic heritability is considered to be an important component of the link between parents’ and children’s family size (see Bernardi 2016). The literature notes two possible dimensions of the genetic component of fertility transmission. First, some people may inherit a health-related constraint on their ability to conceive and, like their mother or father, may be less likely to conceive (Murphy and Knudsen 2002). With the development of efficient contraception methods and assisted medical reproduction, this potential effect of physiological abilities is likely to have diminished over time. However, no inheritance of the physiological ability to conceive has been identified in times without contraception, which suggests that this component plays only a minor role (see the literature review in Murphy 1999).

The second, most commonly cited dimension of genetic heritability is the natural orientation to having children. The idea of a transmission of traits corresponding to a genetic disposition to having children has become increasingly popular among scientists (Kohler et al. 2006; Mills and Tropf 2016; Nisén et al. 2013): fertility behavior may be transmitted from one generation to the next via individual dispositions related to genes (Bernardi 2016; Miller 1992). In addition, the genetic endowment of individuals seems to interact with the environment, directly and through socioenvironmental factors that act on gene expression, as developed in epigenetics (Landecker and Panofsky 2013; Mills and Tropf 2016). Consequently, the heritability of traits becomes contingent on the environment; in particular, the expression of biological variables is stronger when the range of choices is larger (Bras et al. 2013). This corresponds to Udry’s (1996) conjecture that fertility traits have more leeway to express themselves in societies with less social control but are more constrained where norms are strong.

This approach was embraced by important research on genetic heritability based on the Danish twin registry and using an advanced twin design method (Kohler et al. 1999, 2002). This research shows that overall, the genetic influence on family size transmission (including physiological ability and natural orientation) has become more prominent over time. The genetic heritability component is systematically stronger in cohorts with a broader range of life course alternatives (Kohler et al. 1999). The correlation between parents’ and children’s family size due to fertility traits thus seems to be reinforced in contemporary societies.

*Genetic factors predict an increase in the intergenerational transmission of family size across birth cohorts.*

### Socialization

The socialization process based on social learning and social influence through peers is also a major mechanism explaining family size correlation (Bernardi 2016; Kolk 2014). Children observe their parents’ behavior and are thus exposed to intrafamily norms (Booth and Kee 2009) and parental preferences (Axinn et al. 1994). Notably, family size preferences seem to be exerted through social pressure and subjective obligation (Bernardi 2003), which are shaped during childhood. By a mimetic effect or through moral obligations, growing up within a large or small family may thus increase preferences for the same type of family size through a desire to perpetuate the family image instilled during childhood (Lois and Arránz Becker 2013).

The socialization process and its outcomes vary by social background. The transmission of class-based family culture and values was strong at the beginning of the twentieth century (Van Bavel and Kok 2009). Religious groups also have specific socialization models and particularly distinct fertility levels and ideals (Philipov and Berghammer 2007). Consequently, the large changes in the structure of society over the century (in terms of social classes and religion) were certainly crucial in explaining the change in the intergenerational family size correlation. In France, the social groups that shrunk over the century more often had very small families (e.g., employers, including shopkeepers) or very large families (e.g., farmers or manual workers) (Chauvel 2001; Deville 1979). Religious attendance and belief fell steadily between the 1915–1924 and 1945–1954 birth cohorts in the early secularizing societies, such as France (Kaufmann et al. 2012), while practicing Catholic families were often very large (Baudin 2015; Régnier-Loilier and Prioux 2008). We thus expect the drop in the share of families with a small- or large-family culture to reduce fertility transmission, particularly the transmission of extreme family sizes.

In addition, opportunities for social mobility (Glass et al. 1986; Goody 1973), mass education (Breen 2010), and the decline in religious adherence within families (Lehrer and Chiswick 1993) may have weakened the link between parents’ and their children’s family sizes. Notably, an association between upward social mobility and fertility decrease has been observed in many developed countries (see the review in Kasarda and Billy 1985). Desplanques (1985) showed that in France, women who married up had much smaller families than those who married men of the same social background as their father. Likewise, total family size was found to be lower among those with higher education than their parents (Brzozowska 2015).

*The component of the intergenerational transmission related to socialization thus predicts a decrease in the parent-child correlation over time.* W*e also expect that the reproduction of small and large families has shrunk.*

### Data

The Enquête Famille et Logements 2011 (EFL, INED-INSEE) is a survey conducted in association with the French census. It focuses on the family and therefore provides the respondents’ fertility history. The sample covers 360,000 individuals aged 18 or older residing in metropolitan and overseas France who filled out a form distributed along with the census to a representative sample of men and women.^1^ Women were oversampled, however, to provide a sample large enough for a detailed study of fertility histories. Respondents were asked to give their total number of children, including biological, adopted, and deceased children. We have information on the occupation and place of birth of the respondents and their parents, and on the respondents’ level of education and current marital status. A restriction of the survey is that either all adult women or all adult men in the household were interviewed, but never all household members. Consequently, it is not possible to focus on couples. Our study includes all women and men, regardless of their current partnership status and conjugal history.

Our study covers the fertility of individuals born in 1922–1966 in regard to their parents’ fertility, thus capturing almost one century of French fertility. Completed fertility corresponds to the total number of children at the end of the reproductive career.^2^ The study is thus restricted to respondents who have reached age 45, and our results are not sensitive to calendar effects, such as postponement of parenthood. The very large sample size is definitely an asset, but the retrospective and self-reported survey nature is a drawback. Brée et al. (2016) explored the difficulties linked to recall errors and selectivity of mortality in women’s retrospective fertility histories by comparing the French Family Surveys 1982, 1990, 1999, and 2011 as well as data from censuses and civil registration. Despite that the data were self-reported, Brée et al. found reasonable consistency between the data sources for completed fertility, with a small underestimation of a similar magnitude for all cohorts in the 2011 survey: the biases linked to differential mortality and recall errors appeared to be limited for women.^3^

In general, the childbearing histories available for men are reported to be of lesser quality than those for women (Joyner et al. 2012; Mazuy and Lelièvre 2005), notably because in the case of relationship dissolution, men do not live with their young children, but mothers often do. Because of the survey design and the gender gap in life expectancy, the sample size is smaller for older male cohorts than for older female cohorts. Compared with women’s reports, men’s reports of fertility level and number of siblings in older cohorts also show less consistency with respect to the previous 1999 family survey. This suggests that the data for men at advanced ages are more likely to suffer a bias linked to selection or recall error. We thus present the results from only the 1932 to the 1966 birth cohorts for men.

We adopt a definition of the respondent’s number of siblings that includes adopted, half-, deceased, and full siblings. In these cohorts, there were few stepfamilies. We ran robustness checks on a restricted definition excluding half-siblings that might have shared only partially their childhood with the respondent, and the main results were unchanged (for in-depth results on the difference in transmission for full and half-siblings, see Murphy and Knudsen 2002). For the observed cohorts, the number of siblings is consistent with that observed from previous family surveys (conducted in 1982, 1990, and 1999) for both men and women, with a constant small downward bias due to a change in the question.^4^

Our study covers 136,735 women aged 45 to 89 years and 68,331 men aged 45 to 79 years, all of whom were beyond reproductive age at the time of the survey. They are grouped into nine 5-year birth cohorts. Table A1 in the online appendix shows the distribution of the variables used in our analysis. The institute that provides the data (Direction des Statistiques Démographiques et Sociales—INSEE 2014) imputed some of the missing values. Some variables on parents’ socioeconomic background had missing information; the father’s occupational status could be missing for children who grew up with a single mother, for instance. In such a case, we add a category “not available” to capture this paternal absence indirectly.

## Method

The intergenerational transmission of family size is measured by the association between the parents’ fertility and the respondent’s total number of children. First, we calculate both linear and rank correlation indicators. Rank correlation, such as Spearman and Kendall estimates, is less sensitive to extreme values of indicators compared with a linear correlation estimator and is better suited to the discrete nature of the number of children. Linear correlations are also presented for comparison with previous studies that used them.

Second, we implement multivariate models to assess whether there has been a change in the intergenerational family size relationship, net of the fertility changes across cohorts. Our dependent variable is the total number of children, and our main variable of interest is the respondent’s number of siblings. The number of children is a discrete variable, so we apply ordered logistic models or Poisson regressions.^5^ We prefer ordered logistic models throughout our study because parity-specific marginal effects are straightforward to calculate. We present the results of Poisson regressions in the online appendix (see Table A3) because these models are used in many other studies (Baudin 2015; Booth and Kee 2009; Murphy and Wang 2001). The results of the two models are very similar.

We include nine 5-year birth cohorts for women born between 1922 and 1966, and seven 5-year birth cohorts for men born between 1932 and 1966. A coefficient of the interaction between birth cohort and sibship size expresses the strength of the intergenerational relationship over time. It allows us to measure the trend of the intergenerational relationship between the fertility of parents and children, net of the fertility changes that occurred simultaneously over the century.

Third, we move to a parity-specific approach that allows us to measure the strength of the sibship size effect along the fertility distribution. First, descriptive statistics show the distribution of own total number of children by number of siblings across birth cohorts. We then estimate whether sibship size plays a significant role for having zero to seven^6^ children using the marginal effects of the previous ordered logit model for each birth cohort by sex.^7^ The coefficients obtained for the number of siblings and interactions with cohorts in the ordered logistic model refer to the latent process that drives the respondent choice between the categories of family sizes.

In the multivariate approaches, to see whether individual characteristics may interfere in the intergenerational relationship between parents’ and children’s fertility, we systematically perform our regressions with and without controls. In the three adjusted models, we first control only for parent’s available characteristics (social group and mother’s and father’s country of birth) as possible moderators. Second, we control for respondent’s characteristics (country of birth, social group, and educational level). A last model includes both parent’s and respondent’s characteristics. The respondent’s age at first birth is available in the data but, by definition, only for respondents who had children. Because it is important to include childless respondents in order to maintain a broad picture of fertility levels over the century, we do not include age at first birth in our models.

## Results

### Change in Fertility and Number of Siblings

**Fig. 1:**
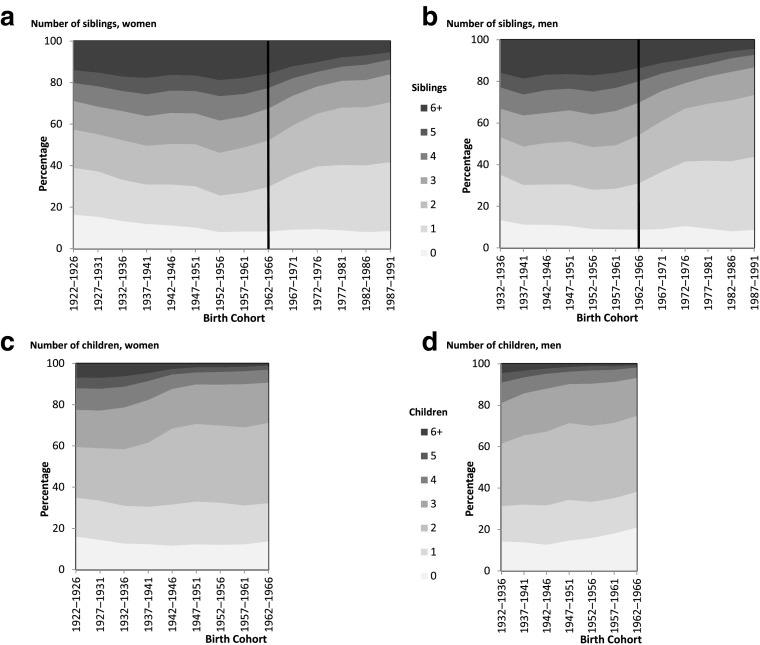
Distribution of number of siblings and of number of children by birth cohorts among women and men. Number of siblings includes adopted, half-, deceased, and full siblings. The sample is women aged 20–89 and men aged 20–79 at the survey for the number of siblings, and women aged 45–89 and men aged 45–79 for the number of children. *Source:* Enquête Famille et Logements 2011 (EFL, INED-INSEE).

Over a long period, the intergenerational correlation of fertility behaviors cannot be disconnected from historical changes in fertility. Over the century, the share of large families (those with three or more children) decreased in France, and the two-child family became predominant: when comparing women born in the 1930s with those born in the 1950s onward, the probability of having two children rose from 3 in 10 to 4 in 10 (Fig. 1, panel c). Men displayed similar patterns, although the rise of the two-child family started in older cohorts and was more gradual (Fig. 1, panel d).

The distribution of the number of children changed quickly across the 1922–1966 birth cohorts, but the number of siblings remained quite stable for the cohorts studied except in the latest cohorts (Fig. 1, panels a and b). Minor changes consisted of a small decrease in the number of individuals with only one sibling until the late Baby Boom cohorts. Beginning with the 1957–1961 birth cohorts, originating from a two-child family became more and more frequent: only in this second stage did the two-child family become the norm for both parents and children.

**Fig. 2:**
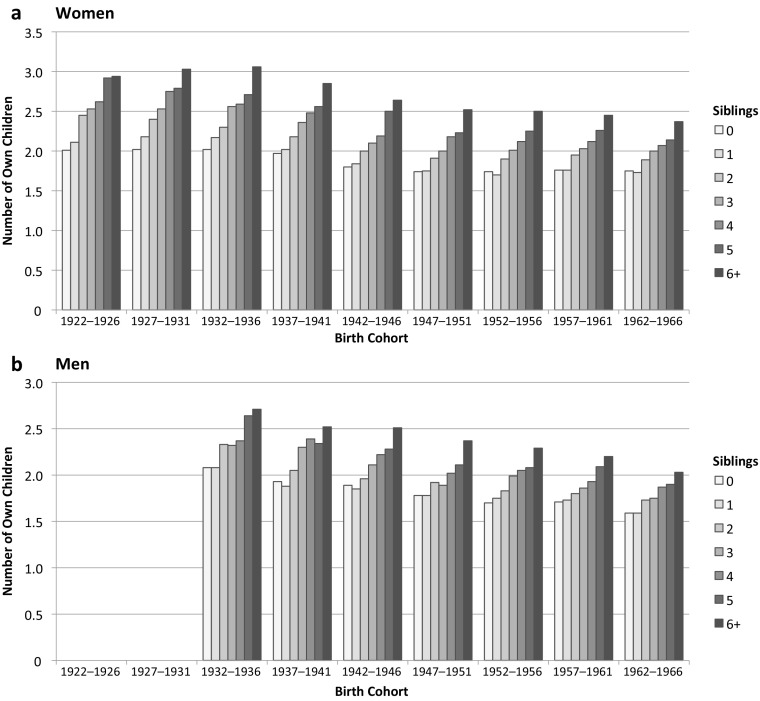
Mean number of own children by number of siblings among women and men, by birth cohort. The sample is women aged 45–89 at survey and men aged 45–79 at survey. *Source:* Family Survey 2011 (EFL, INED-INSEE).

Figure 2 shows the clear positive association between parents’ and children’s family size. The mean number of children increased steadily with the number of siblings in the family of origin for both men and women. As time passed, the magnitude of the contrast between those with few siblings and those with many siblings decreased considerably. Only men and women from very large families (those with six or more children) had proportionally even more children consistently across cohorts.

For women, the fertility of only-children still differed from the fertility of one-sibling respondents in the first half of the century, whereas recently it has become similar. For men, the fertility of these two groups appears similar across all cohorts, suggesting that having zero or one sibling is almost the same. Murphy and Knudsen (2002) found a similar result for both men and women from a recent cohort in Denmark (1968–1969): fertility was equivalent whether there was zero or one sibling, but above this number, fertility increased with each additional sibling.

### Correlations

Table 2 presents the linear and rank correlations between male and female respondents and their parents’ fertility, by sex and birth cohort. The simple linear correlation remains positive and significant, at levels comparable with those observed in previous studies: .17 for women and .13 for men. The stronger correlation for women than for men is also confirmed. The two other indicators of rank correlation, Spearman and Kendall, are also higher for women than for men.^8^ Women still surpass men in adopting fertility levels that conform to those of their parents.

**Table 2:** Linear and rank correlations of the number of children and number of siblings, by sex and birth cohort

	Linear		Spearman		Kendall		*N*	
Birth Cohort	Women	Men	Women	Men	Women	Men	Women	Men
All (1932–1966)	.175	.128	.167	.121	.131	.095	119,910	62,176
1922–1926	.147		.164		.129		6,573	
1927–1931	.167		.161		.123		10,252	
1932–1936	.176	.123	.158	.110	.123	.085	12,059	5,367
1937–1941	.187	.136	.172	.134	.134	.107	12,151	6,024
1942–1946	.191	.146	.183	.144	.143	.112	14,449	7,601
1947–1951	.186	.143	.180	.129	.142	.102	20,075	10,505
1952–1956	.188	.131	.178	.129	.140	.100	19,770	10,470
1957–1961	.166	.112	.166	.110	.131	.087	20,318	10,848
1962–1966	.153	.111	.147	.102	.117	.080	21,088	11,361
*N*							136,735	62,176

*Note:* The sample is women aged 45–89 at survey and men aged 45–79 at survey.

*Source:* Enquête Famille et Logements 2011 (EFL, INED-INSEE).

The two indicators based on rank correlation (considered to be the most appropriate, as mentioned in the Method section) both display similar patterns of correlation over time. The correlation between number of children and number of siblings decreased very slowly until the mid-1930s cohorts. There was then a major reshuffling of family size distribution, and the correlation strengthened for both sexes, reaching a peak for the cohorts born during World War II (birth cohorts 1932–1946).^9^ From then on, Baby Boomers initiated the second demographic transition. They modified their family and fertility behavior with respect to their parents’ generation, which may explain the substantially decreasing correlations for both men and women. However, correlation measures the intensity of the relationship between two variables without assuming a direction of causality. In our case, regressions are more suitable because the child’s fertility level may depend on that of the parents, but the reverse is not possible. Moreover, regressions may include several covariates. We thus implement multivariate models.

### Multivariate Models

**Fig. 3:**
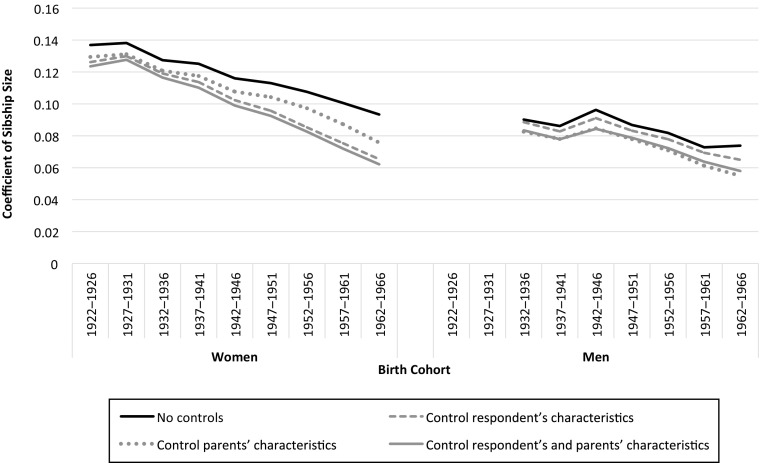
Results of ordered logistic regression of completed fertility for women and men and for various adjustment variables: coefficient of sibship size by birth cohort. Additional adjustment variables are respondent’s level of education, country of birth, occupational status, mother’s and father’s country of birth, and their occupational status (see online appendix C). The sample is women aged 45–89 and men aged 45–79 at survey. In the 1922–1926 birth cohort and with all controls, for a one-unit increase in the number of siblings, the number of children is expected to change by 0.117 + 0.007 = 0.124 (see Model 4 for women in Table 3) in the log-odds scale, while holding the other variables in the model constant. Full results are available in Table A2 in the online appendix. *Source:* Enquête Famille et Logements 2011 (EFL, INED-INSEE).

Figure 3 shows the coefficients of the interaction terms between number of siblings and birth cohorts of the ordered logistic model, starting without covariates and adding controls by groups. Table 3 presents the coefficients and standard errors for these variables of interest depending on the group of control variables introduced (Models 1–4). Table A2 in the online appendix shows the full set of coefficients and standard errors for all covariates. An alternative model using a Poisson model is provided in Table A3.

**Table 3:** Coefficients from ordered logit of completed fertility, by sex

	Women				Men			
	Model 1	Model 2	Model 3	Model 4	Model 1	Model 2	Model 3	Model 4
Total Number of Siblings	0.127***	0.119***	0.121***	0.117***	0.090***	0.089***	0.083***	0.083***
	(0.006)	(0.006)	(0.006)	(0.006)	(0.010)	(0.010)	(0.010)	(0.010)
Respondent’s Birth Cohort (ref. = 1932–1936)								
1922–1926	–0.091*	–0.123**	–0.089*	–0.122**				
	(0.043)	(0.043)	(0.043)	(0.043)				
1927–1931	–0.055	–0.082*	–0.058	–0.084*				
	(0.038)	(0.038)	(0.038)	(0.038)				
1937–1941	–0.131***	–0.066^†^	–0.121***	–0.064^†^	–0.148**	–0.163**	–0.154**	–0.164**
	(0.036)	(0.036)	(0.036)	(0.036)	(0.052)	(0.052)	(0.052)	(0.052)
1942–1946	–0.314***	–0.201***	–0.296***	–0.201***	–0.289***	–0.301***	–0.286***	–0.297***
	(0.034)	(0.034)	(0.034)	(0.034)	(0.049)	(0.050)	(0.050)	(0.050)
1947–1951	–0.421***	–0.293***	–0.405***	–0.301***	–0.395***	–0.400***	–0.399***	–0.403***
	(0.032)	(0.032)	(0.032)	(0.032)	(0.046)	(0.047)	(0.046)	(0.047)
1952–1956	–0.431***	–0.289***	–0.417***	–0.304***	–0.398***	–0.395***	–0.396***	–0.396***
	(0.033)	(0.033)	(0.033)	(0.033)	(0.047)	(0.047)	(0.047)	(0.047)
1957–1961	–0.368***	–0.175***	–0.345***	–0.190***	–0.448***	–0.453***	–0.446***	–0.452***
	(0.032)	(0.033)	(0.033)	(0.033)	(0.047)	(0.047)	(0.047)	(0.047)
1962–1967	–0.401***	–0.161***	–0.370***	–0.182***	–0.581***	–0.570***	–0.572***	–0.569***
	(0.032)	(0.033)	(0.032)	(0.033)	(0.046)	(0.047)	(0.047)	(0.047)
Interaction Birth Cohort and Total Number of Siblings (ref. = 1932–1936)								
Siblings × 1922–1926	0.009	0.007	0.009	0.007				
	(0.011)	(0.011)	(0.011)	(0.011)				
Siblings × 1927–1931	0.011	0.011	0.010	0.011				
	(0.010)	(0.010)	(0.010)	(0.010)				
Siblings × 1937–1941	–0.002	–0.005	–0.003	–0.006	–0.004	–0.006	–0.005	–0.006
	(0.009)	(0.009)	(0.009)	(0.009)	(0.013)	(0.013)	(0.013)	(0.013)
Siblings × 1942–1946	–0.011	–0.017*	–0.013	–0.018*	0.006	0.002	0.002	0.001
	(0.008)	(0.008)	(0.008)	(0.008)	(0.012)	(0.012)	(0.012)	(0.012)
Siblings × 1947–1951	–0.014^†^	–0.023**	–0.017*	–0.024**	–0.003	–0.005	–0.005	–0.005
	(0.008)	(0.008)	(0.008)	(0.008)	(0.012)	(0.012)	(0.012)	(0.012)
Siblings × 1952–1956	–0.020*	–0.034***	–0.024**	–0.034***	–0.008	–0.011	–0.012	–0.011
	(0.008)	(0.008)	(0.008)	(0.008)	(0.012)	(0.012)	(0.012)	(0.012)
Siblings × 1957–1961	–0.027***	–0.044***	–0.034***	–0.045***	–0.017	–0.019	–0.021^†^	–0.020^†^
	(0.008)	(0.008)	(0.008)	(0.008)	(0.012)	(0.012)	(0.012)	(0.012)
Siblings × 1962–1967	–0.034***	–0.054***	–0.045***	–0.054***	–0.016	–0.024*	–0.028*	–0.025*
	(0.008)	(0.008)	(0.008)	(0.008)	(0.012)	(0.012)	(0.012)	(0.012)
*N*	136,735	136,735	136,735	136,735	62,176	62,176	62,176	62,176
Controls for Characteristics:								
Of respondent	No	Yes	No	Yes	No	Yes	No	Yes
Of parents	No	No	Yes	Yes	No	No	Yes	Yes

*Notes:* The sample is women aged 45–89 at survey and men aged 45–79 at survey. Standard errors are shown in parentheses. Interpretation: For a one-unit increase in the number of siblings, the number of children is expected to change by 0.117 (Model 4 for women) in the ordered log-odds scale when the other variables in the model are held constant. Full results are available in Table A2 in the online appendix.

*Source:* Enquête Famille et Logements 2011 (EFL, INED-INSEE).

^†^*p* < .10; *p < .05; **p < .01; ***p < .001

In the basic model, which includes only birth cohort and the interaction between birth cohort and number of siblings, we observe a decreasing trend in the relationship between sibship size and number of children for women. Thus, net of the fertility decline across cohorts, the strength of the relationship between daughters’ and their parents’ fertility weakened gradually (significantly for the last five birth cohorts relative to the 1932–1936 birth cohort). For men, the rebound observed in the previous basic correlations is still observed, followed by a downward trend from the first postwar birth cohort. However, the drop is less marked than for women and is barely significant.

When we include parents’ characteristics in the model (mother’s and father’s country of birth and occupation), the intergenerational link between the parents’ and respondent’s number of children weakens for both men and women and across all generations. Controlling for parental characteristics thus attenuates the intergenerational fertility relationship, although we do not know whether they act directly or as moderators. On the other hand, when we include controls for the respondents’ characteristics, a contrast between men and women arises: while the level of intergenerational fertility transmission remains about the same among men, the addition of these individual characteristics substantially reduces the strength of the association among women in all birth cohorts. This indicates greater heterogeneity among women than men in their relationship to the parental model of family size. This result also emphasizes the important role of education in fertility for women relative to men: the spectacular increase in educational attainment in the second half of the twentieth century concerned women in particular.

Finally, after both groups of variables are introduced simultaneously in the last model, the coefficients for women drop only slightly more than the strong decrease observed when we control for individual characteristics only: characteristics specific to parents do not account for much in the overall association. This result also confirms the heterogeneity in women’s response to family size of origin. Among men, however, it is mainly the introduction of parental characteristics in the model that lessens the intergenerational link.

Overall, the negative slope in the correlation across birth cohorts persists whatever the group of variables introduced. After controls are included, the association with parents’ fertility is halved for women from the cohorts born in the 1920s to those born in the 1960s. For men, however, the drop is much more recent and is approximately one-third. It seems that women have distanced themselves more than men from their family’s fertility behavior since the cohorts born around 1930 (cohorts in which large numbers of women started working).

To this point, we have measured the average statistical effect of sibship size on offspring. Yet, the intergenerational relationship varies along the fertility distribution.

**Fig. 4:**
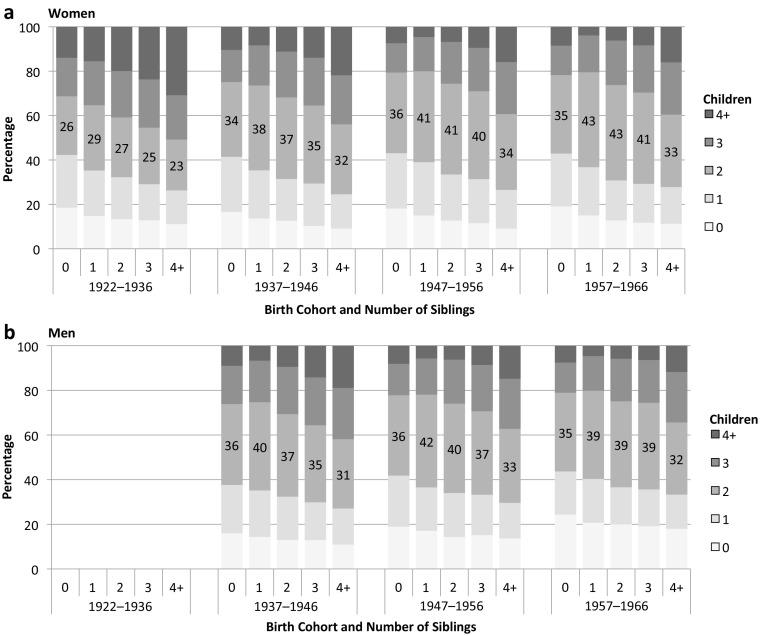
Family size distribution by birth cohort and number of siblings among women and men. The sample is women aged 45–89 and men aged 45–79 at survey. *Source:* Enquête Famille et Logements 2011 (EFL, INED-INSEE).

In Fig. 4, we represent the family size distribution by number of siblings, for each birth cohort and sex. This shows that respondents from small families (without siblings) tend to have few children, and those from large families (with three or more siblings) tend to have large families, with an almost linear relationship. Roughly 40 % of respondents without siblings remain childless or have an only-child regardless of the birth cohort and sex, compared with one-quarter of those with four or more siblings. Complementarily, in the 1957–1966 birth cohorts, one-fifth of only-children have three or more children, compared with 55 % to 60 % of those with four or more siblings. The figure also emphasizes the diffusion of the two-child families in cohorts born from 1937 onward, with more than 40 % of respondents born after the World War II having exactly two children. Although the diffusion concerns all respondents, it is more massive among those having one to three siblings, whereas only-children and respondents who grew up in large families are less likely to adopt the norm because of their preferences for, respectively, small and large families. Finally, in the 1922–1936 birth cohorts, women coming from large families preferred four children or more: over 25 % of women with three or more siblings had four or more children. In the most recent cohorts (1957–1966), people with three or more siblings rather have three or more children. This suggests that the third child has become the “extra child,”—that is, the child who increases the family size beyond the prevalent norm of the cohort—and is taken up mostly by those coming from large families.

**Fig. 5:**
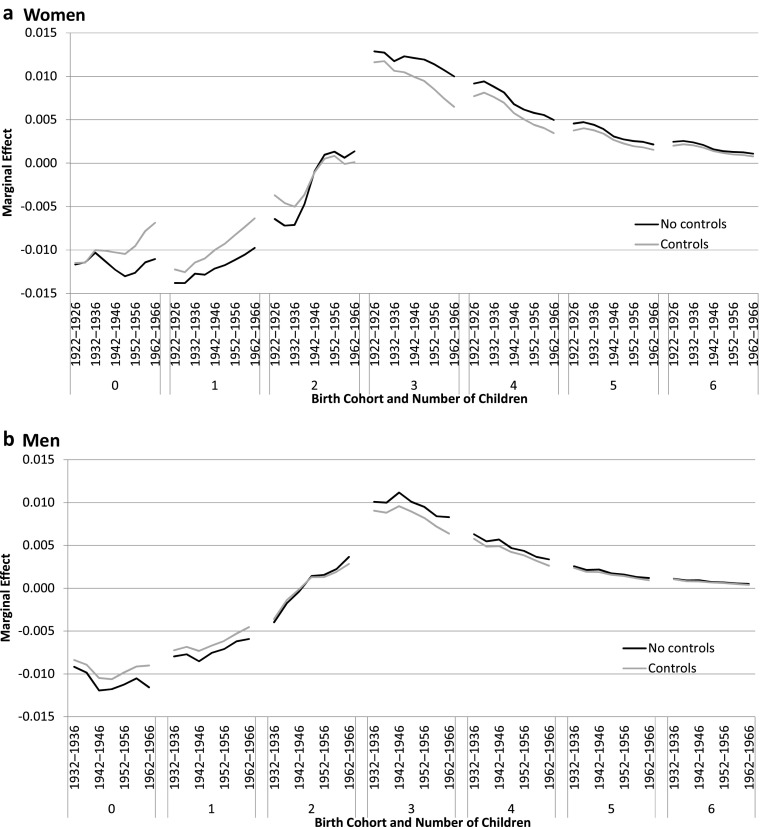
Marginal effect of the origin family size for number of children and birth cohort among women and men (ordered logistic model), without controls and with all controls (Models 1 and 4 of Table A2 in the online appendix). The value presented is the average change in predicted probabilities for a small change of the number of siblings. The sample is women aged 45–89 and men aged 45–79 at survey. Adjustment variables included are level of education, country of birth, occupational status, mother’s and father’s country of birth, and their occupational status. *Source:* Enquête Famille et Logements 2011 (EFL, INED-INSEE).

To explore across cohorts which family sizes are most sensitive to the original family size, we use ordered logit regressions performed by sex, where total family size is an outcome from 0 to 12 and more children. The coefficient of number of children rises with the number of siblings (Table 3). We display in Fig. 5 the marginal effect of having more siblings^10^ on achieving each parity from 0 (childless) to 6. The closer to zero the marginal effect is, the less influence a small change in the number of siblings has on own family size. (See also Fig. A1 in the online appendix, which presents the predicted probabilities for different sibship sizes and helps with the interpretation of the results.) The marginal effect of having more siblings increases up to three children. At higher parities, the marginal effect lessens. The family size most sensitive to a marginally larger sibship size is the three-child family.

Regarding trends, family size of origin lost some of its positive impact on the reproduction of large families across the 1922–1971 birth cohorts for women, but it remained essential to explain families of three or four children (Fig. 5). The negative impact of a larger sibship size on families with zero and one child remained strong across cohorts. Figure A1 in the online appendix helps interpret this result: at first, women coming from large families were less likely to have two children. With the spread of the normative model of the two-child family, sibship size lost all its influence on this particular family size: women forming two-child families were stemming from all sibship sizes. The contrasting persistence of the influence of the number of siblings for having a third child reinforces the idea that the third child has increasingly become an “extra child” favored by people from large families. Among men, we observe similar results, with the strongest effects of a marginal change in the number of siblings for the small parities (zero and one child) and for parity 3, and a decreasing effect across birth cohorts. As among women, the influence of sibship size on forming a two-child family is small for men; the marginal effects are close to zero. Thus, a relative change in the number of siblings only slightly affects the probability to have a two-child family, whereas the same change increases the probability of having three children to a much greater extent. We observe that having two children became a little more frequent for men coming from large families in the recent cohorts. If such a trend persists and extends to women, the second child may become the new “extra child” in the future in a context of fertility decline.

The models including controls lead to roughly the same conclusions. Overall, small and large families became less sensitive to the family size of origin across birth cohorts after controls are included. (In families of five of more children, the effect of the inclusion of covariates is not visible because the levels are already close to zero.) For all family sizes other than two, the magnitude of the sibship size effect was thus partly driven by socioeconomic factors. Among women, downward trends are reinforced across birth cohorts after controls are included, so that the effect of sibship size for all parities was by far the weakest in the recent birth cohorts. Among men, the inclusion of socioeconomic controls diminished the magnitude but did not change the time trend much.

## Conclusion and Discussion

With the intergenerational correlation between fertility levels well established, our study offers a more in-depth examination of the strength of this link in the long run. First, we confirm a persistent positive link between parents’ and children’s fertility in France, demonstrating that the family size of origin is still a determinant in fertility decisions. The magnitude of the correlation is comparable with that observed in other countries, but the trend differs. The strength of the relationship decreased over the long period studied, particularly for cohorts born since the mid-1950s. After we included controls for socioeconomic factors, the relationship with the size of the family of origin was twice as strong for women born in the late 1920s as for those born in the 1960s. This result is based on a large data set with several cohorts observed at the end of their reproductive age, thus giving it particular strength.

Most previous post-transitional studies in developed societies have not found a decrease but rather stability or an increase in cohorts born in the middle of the century (Dahlberg 2013; Murphy and Wang 2001) and stability later on (Murphy 2013). These results could contrast with ours because of methodology discrepancies (e.g., they used fewer cohorts that have not always completed their fertility; observations are sometimes based on small samples; confounding factors are not always controlled for). Considering this study in the French context, however, is important because this country may have been a forerunner in the downward trend of the intergenerational transmission of fertility. The country has benefited from very generous family policies for decades and has great tolerance for the issue of parents’ marital status: couples can decide quite freely whether to have children in a consensual union, in a PACS (civil partnership), or within marriage. This family-friendly context probably allows people to make decisions about their family size with more freedom and independence than elsewhere, although in such a family-oriented country, this could also translate into more social pressure to have children. Therefore, it is important in the long run to replicate this study in other countries in order to see whether the weakening of family fertility transmission can also be observed elsewhere.

Which of our expectations regarding the direction of the trend correspond best to our results? The decrease in the intergenerational fertility correlation suggests that in these cohorts and in this context, the weakening of parental influence—generally attributed to the delay in age at childbearing and to social mobility—may have played a greater role than genetic heritability, which rather predicted an increase in the correlation. We thus suggest that the loss of the family’s implicit or explicit power in shaping fertility decisions has led to less transmission. The correlation may have weakened because births have been postponed to a later age, when men and women are less influenced by their family of origin. Religiosity, which we unfortunately could not control for (particularly Catholicism, the dominant religion in France), was probably another key instrument in perpetuating large families in the past, and its decline could explain some of the weaker relationship observed in most recent cohorts (Glass et al. 1986; Lehrer and Chiswick 1993). Genetic heritability may still have pushed the correlation up across cohorts, but the effects previously described have counterbalanced its influence.

Second, an original aspect of the present study is that it goes beyond the standard average relationship and looks at the overall distribution of fertility to question the adherence to the norm in terms of family size and transmission among small and large families. We observe that the correlation is driven mainly by small and three-child families. As expected, the intergenerational correlation disappeared among two-child families as people started to adopt this family size regardless of their number of siblings. However, the correlation also weakened at most parities, in line with the argument of a diminishing role of socialization in shaping fertility levels. Sensitivity to origin family size remained strong among childless women and men, possibly because that group still strongly differs from the *Zeitgeist* in France. Three-child families remain very positively influenced by the size of the family of origin. Coming from a large family thus seems to play a particular role in having an “extra” third child.

Socioeconomic characteristics could act either directly on the number of children as mediating factor in the relationship between parental family size and child family size (own characteristics) or indirectly as moderator in this relationship (parental characteristics). Although our models do not allow an empirical test of the role of these variables in the relationship between parents’ and own fertility, we observe that the association between parental and own family size weakens after major socioeconomic factors are included and that the decreasing trend over time persists. This is the case for the overall correlation as well as for having small and three-child families, suggesting that the perpetuation of family sizes may be the choice of specific educational and social groups. The fact that introducing covariates for two-child families has little effect reinforces the impression that this is a pivotal category that includes people from all backgrounds and from all family sizes.

Third, we observe a gender convergence of the strength of intergenerational transmission across cohorts. In the 1932–1966 birth cohorts, the size of the family of origin was more strongly associated with the number of own children among women compared with men. The much faster decrease among women, particularly after we control for basic sociodemographic characteristics, signals a convergence between sexes. The convergence is in line with results showing a possible shift toward men taking a role similar to women in couples’ fertility decisions (Bauer and Kneip 2013). This interpretation is in line with Fasang and Raab’s (2014) finding that educational upward mobility, particularly strong for women over the period studied, reduced the probability that the children’s life courses would closely resemble those of their parents. Yet, our results show much more heterogeneity in women’s response to the family model than in men’s: some socioeconomic groups of women are more independent of the parental family model than others.

Several limitations to this study must be mentioned. Although the large sample size and retrospective nature of the database are great advantages, the data remain incomplete in other respects, potentially limiting the scope of the present study. First, we do not control for parenthood timing: neither respondent’s age at first birth (because the present study includes childless people) nor age of respondent’s mother at first birth (which are not available in the data) (Barber 2001; Riise et al. 2016). Fertility timing itself is exposed to influences from parental background (Riise et al. 2016; Steenhof and Liefbroer 2008). In addition, the birth order of the respondents, also known to be related to fertility transmission, is not available (Murphy and Knudsen 2002). Second, we cannot explore the gender difference further because we are unable to control simultaneously for the size of both partners’ families of origin. Previous studies, notably Booth and Kee (2009), found that the origin family size of both the wife and husband matter. Third, the definition of siblings used is quite broad and includes adopted, half-, deceased, and full siblings. Even if the cohorts born before the 1970s did not often have half-siblings (approximately 7 % had half-siblings in the cohorts studied), the correlation between half-siblings and own fertility could be weaker because they did not necessarily grow up in the same household as the respondent. However, robustness checks excluding half-siblings gave very similar results. The choice of definition of sibship and whether to include half-siblings might become more important in subsequent cohorts, given the strong increase in the number of stepfamilies. Finally, apart from the aforementioned robustness checks, we are not able to further control for children’s and parents’ separation or marital status, which are both potentially endogenous and could alter the degree of intergenerational reproduction of fertility behaviors (Axinn and Thornton 1996; Kalmijn 2015). Divorce and separation, in particular, increase the prevalence of small families for both sexes (and, slightly, of large families for men) (Beaujouan 2010; Jansen et al. 2009). Because those whose parents divorced are more likely to divorce (Lyngstad and Jalovaara 2010), the increase in divorce for recent cohorts may be a factor in the transmission among small families.

All in all, through the analysis of the relationship between parents’ and children’s fertility, this study provides insights into the current transformations of fertility. If the positive link between an individual’s number of siblings and her/his own fertility is weaker today, it is probably because the cultural habits of the family of origin have become less important to generations born in the second half of the twentieth century. The increasing emphasis on individuality during the second demographic transition already suggested that family background had become less prominent. Other demographic events also became less linked to the family of origin. For instance, the link between parental divorce and that of children appears to be weaker now than in earlier generations that had stronger marriage norms and barriers to divorce (Wagner and Weiss 2006). Whereas family background is losing its importance, the achieved level of fertility might depend more today on economic factors, especially in a context of economic crisis in which the dual-earner family is widespread (Balbo et al. 2013). This possible transition in developed countries from family drivers of fertility to economic ones reinforces the need to give more support to public policies that reduce economic inequalities and that assist individuals in their family formation process.

## Electronic supplementary material

ESM 1 (PDF 221 kb)

## Footnotes

1 Information on the survey methods is available online at http://www.insee.fr/en/methodes/default.asp?page=sources/ope-enq-famille-logements.htm.

2 The fertility rate among men aged 45 and older has been 0.08 children per man since the 1990s—that is, 4 % of the total fertility rate (Prioux 2005).

3 A recent study in a high-income country, Sweden, showed almost no effect of the number of siblings on mortality risk (Baranowska-Rataj et al. 2017). Thus, the population surveyed (i.e., those who survived) should have the same number of siblings as the original population. However, for the oldest cohorts, childless individuals or those who had several children are more likely to be prematurely dead and thus not observed (Barclay et al. 2016). The fertility distribution might be slightly skewed for this reason, and results may be affected by a small bias, limited here.

4 Unfortunately, the previous surveys could not be used in conjunction with the 2011 survey because of a change in question wording and consequently in the overall number of siblings reported. Before 2011, respondents were asked for their mother’s number of children; in 2011, they were asked for their number of siblings (including stepbrothers and stepsisters from either parent).

5 The conditions of the Poisson regression, variance equal to the mean, are satisfied. For women, the mean is 2.16, and the variance is 2.27. For men, the mean is 1.99, and the variance is 2.01. Our data are not affected by underdispersion, so a Poisson regression is appropriate.

6 The ordered logit model is estimated from 0 to 12 children and more, but only the marginal effects for probabilities 0 to 7 are represented for the sake of simplicity. Marginal effects of higher parities are negligible.

7 The results of our models are generally consistent with those of an alternative model using quantile regression for discrete data, as exposed in Beaujouan and Solaz (2016).

8 The absolute levels of the different indicators are not directly comparable.

9 Contrary to Fig. 2, which represents the mean number of own children as a function of the number of siblings, the correlation indices take into account the frequency of pairs of both parents’ and respondents’ family sizes: the correlations increased simultaneously with the reshuffling of respondents’ family size observed in Fig. 1.

10 Given that the variable of interest is continuous, the marginal effect represents the change of probability of reaching each family size with a unit increase in the number of siblings.
