# Supplementary material for: Is the Family Size of Parents and Children Still Related? Revisiting the Cross-Generational Relationship Over the Last Century
Source: Demography. 2019 Mar 13;56(2):595–619. doi: 10.1007/s13524-019-00767-5 (PMC6449311; doi:10.1007/s13524-019-00767-5)
Supplement: Supplementary file 1 — (PDF 221 kb) [file 13524_2019_767_MOESM1_ESM.pdf]

Online Appendix

Is The Family Size of Parents and Children Still Related? Revisiting the Cross-Generational Relationship over the Last Century

Eva Beaujouan[1] and Anne Solaz[2]

Eva Beaujouan (corresponding author)
Eva.Beaujouan@oeaw.ac.at

[1] Wittgenstein Centre for Demography and Global Human Capital / Vienna University of Economics and Business (WU), Department of Socioeconomics, Building D4, 3. floor, Welthandelsplatz 1, 1020 Vienna.

[2] Institut national d'études démographiques (INED), F-75020 Paris, France.

Appendix: Family Size of Parents and Children

Table A1: Descriptive statistics of the covariates used in the study

Variable		Women Mean/ distribution	Women Std. Dev.	Women Min	Women Max	Men Mean/ distribution	Men Std. Dev.	Men Min	Men Max
**Total number of children**		2.16	1.51	0	11	1.97	1.38	0	11
**Birth cohort respondent**	1922–1926	4.8	0.21	0	1				
	1927–1931	7.5	0.26	0	1				
	1932–1936	8.8	0.28	0	1	8.6	0.28	0	1
	1937–1941	8.9	0.28	0	1	9.7	0.30	0	1
	1942–1946	10.6	0.31	0	1	12.2	0.33	0	1
	1947–1951	14.7	0.35	0	1	16.9	0.37	0	1
	1952–1956	14.5	0.35	0	1	16.8	0.37	0	1
	1957–1961	14.9	0.36	0	1	17.4	0.38	0	1
	1962–1966	15.4	0.36	0	1	18.3	0.39	0	1
**Total number of siblings**		3.21	2.71	0	17	3.16	2.66	0	17
**Respondent's level of education**	Lower secondary	48.6	0.50	0	1	33.8	0.47	0	1
	Upper secondary	34.2	0.47	0	1	46.1	0.50	0	1
	Tertiary	17.2	0.38	0	1	20.1	0.40	0	1
**Respondent's country of birth**	France	88.0	0.33	0	1	86.8	0.34	0	1
	Southern Europe	2.8	0.16	0	1	3.0	0.17	0	1
	Rest of Europe	1.7	0.13	0	1	1.6	0.13	0	1
	North Africa	4.6	0.21	0	1	5.5	0.23	0	1
	Rest of the world	2.9	0.17	0	1	3.1	0.17	0	1
**Respondent's occupational status**	Never worked	13.0	0.34	0	1	6.6	0.25	0	1
	Farmer	4.3	0.20	0	1	4.4	0.21	0	1
	Independent	5.0	0.22	0	1	10.0	0.30	0	1
	White collar	6.6	0.25	0	1	16.1	0.37	0	1
	Blue collar	18.4	0.39	0	1	19.3	0.39	0	1
	Clerk	40.1	0.49	0	1	11.0	0.31	0	1
	Manual worker	12.6	0.33	0	1	32.6	0.47	0	1
**Mother's occupational status**	Never worked	37.1	0.48	0	1	38.7	0.49	0	1
	Farmer	11.5	0.32	0	1	10.5	0.31	0	1
	Independent	5.9	0.24	0	1	5.3	0.22	0	1
	White collar	0.8	0.09	0	1	0.9	0.09	0	1
	Blue collar	4.4	0.20	0	1	4.8	0.21	0	1
	Clerk	15.7	0.36	0	1	16.0	0.37	0	1
	Manual worker	10.1	0.30	0	1	8.9	0.28	0	1
	Not available	14.6	0.35	0	1	15.0	0.36	0	1
**Mother's country of birth**	France (or NA)	83.4	0.37	0	1	82.1	0.38	0	1
	Southern Europe	5.2	0.22	0	1	5.4	0.23	0	1
	Rest of Europe	3.1	0.17	0	1	3.0	0.17	0	1
	North Africa	5.0	0.22	0	1	6.1	0.24	0	1
	Rest of the world	3.2	0.18	0	1	3.4	0.18	0	1
**Father's occupational status**	Never worked	0.8	0.09	0	1	0.8	0.09	0	1
	Farmer	14.8	0.36	0	1	13.8	0.35	0	1
	Independent	10.8	0.31	0	1	10.6	0.31	0	1
	White collar	6.2	0.24	0	1	6.8	0.25	0	1
	Blue collar	7.1	0.26	0	1	7.7	0.27	0	1
	Clerk	8.1	0.27	0	1	8.3	0.28	0	1
	Manual worker	33.2	0.47	0	1	32.8	0.47	0	1
	Not available	19.0	0.39	0	1	19.2	0.39	0	1
**Father's country of birth**	France (or NA)	82.6	0.38	0	1	81.3	0.39	0	1
	Southern Europe	5.9	0.24	0	1	6.1	0.24	0	1
	Rest of Europe	3.1	0.17	0	1	3.0	0.17	0	1
	North Africa	5.1	0.22	0	1	6.2	0.24	0	1
	Rest of the world	3.3	0.18	0	1	3.4	0.18	0	1
N		136,735				62,176			

Table A2: Ordered logit of completed fertility, by sex (Coefficients, standard error in parenthesis)

	**Women**				**Men**			
	Model 1	Model 2	Model 3	Model 4	Model 1	Model 2	Model 3	Model 4
**Total number of siblings**								
	0.127***	0.119***	0.121***	0.117***	0.090***	0.089***	0.083***	0.083***
	(0.006)	(0.006)	(0.006)	(0.006)	(0.010)	(0.010)	(0.010)	(0.010)
**Birth cohort respondent (ref = 1932–36)**								
1922–1926	-0.091*	-0.123**	-0.089*	-0.122**				
	(0.043)	(0.043)	(0.043)	(0.043)				
1927–1931	-0.055	-0.082*	-0.058	-0.084*				
	(0.038)	(0.038)	(0.038)	(0.038)				
1937–1941	-0.131***	-0.066†	-0.121***	-0.064†	-0.148**	-0.163**	-0.154**	-0.164**
	(0.036)	(0.036)	(0.036)	(0.036)	(0.052)	(0.052)	(0.052)	(0.052)
1942–1946	-0.314***	-0.201***	-0.296***	-0.201***	-0.289***	-0.301***	-0.286***	-0.297***
	(0.034)	(0.034)	(0.034)	(0.034)	(0.049)	(0.050)	(0.050)	(0.050)
1947–1951	-0.421***	-0.293***	-0.405***	-0.301***	-0.395***	-0.400***	-0.399***	-0.403***
	(0.032)	(0.032)	(0.032)	(0.032)	(0.046)	(0.047)	(0.046)	(0.047)
1952–1956	-0.431***	-0.289***	-0.417***	-0.304***	-0.398***	-0.395***	-0.396***	-0.396***
	(0.033)	(0.033)	(0.033)	(0.033)	(0.047)	(0.047)	(0.047)	(0.047)
1957–1961	-0.368***	-0.175***	-0.345***	-0.190***	-0.448***	-0.453***	-0.446***	-0.452***
	(0.032)	(0.033)	(0.033)	(0.033)	(0.047)	(0.047)	(0.047)	(0.047)
1962–1967	-0.401***	-0.161***	-0.370***	-0.182***	-0.581***	-0.570***	-0.572***	-0.569***
	(0.032)	(0.033)	(0.032)	(0.033)	(0.046)	(0.047)	(0.047)	(0.047)
**Interaction birth cohort and total number of siblings (ref = 1932–36)**								
Siblings*1922–1926	0.009	0.007	0.009	0.007				
	(0.011)	(0.011)	(0.011)	(0.011)				
Siblings*1927–1931	0.011	0.011	0.010	0.011				
	(0.010)	(0.010)	(0.010)	(0.010)				
Siblings*1937–1941	-0.002	-0.005	-0.003	-0.006	-0.004	-0.006	-0.005	-0.006
	(0.009)	(0.009)	(0.009)	(0.009)	(0.013)	(0.013)	(0.013)	(0.013)
Siblings*1942–1946	-0.011	-0.017*	-0.013	-0.018*	0.006	0.002	0.002	0.001
	(0.008)	(0.008)	(0.008)	(0.008)	(0.012)	(0.012)	(0.012)	(0.012)
Siblings*1947–1951	-0.014†	-0.023**	-0.017*	-0.024**	-0.003	-0.005	-0.005	-0.005
	(0.008)	(0.008)	(0.008)	(0.008)	(0.012)	(0.012)	(0.012)	(0.012)
Siblings*1952–1956	-0.020*	-0.034***	-0.024**	-0.034***	-0.008	-0.011	-0.012	-0.011
	(0.008)	(0.008)	(0.008)	(0.008)	(0.012)	(0.012)	(0.012)	(0.012)
Siblings*1957–1961	-0.027***	-0.044***	-0.034***	-0.045***	-0.017	-0.019	-0.021†	-0.020†
	(0.008)	(0.008)	(0.008)	(0.008)	(0.012)	(0.012)	(0.012)	(0.012)
Siblings*1962–1967	-0.034***	-0.054***	-0.045***	-0.054***	-0.016	-0.024*	-0.028*	-0.025*
	(0.008)	(0.008)	(0.008)	(0.008)	(0.012)	(0.012)	(0.012)	(0.012)
**Level of education respondent (ref = lower secondary)**								
Upper secondary		-0.239***		-0.234***		-0.005		0.001
		(0.012)		(0.012)		(0.017)		(0.017)
Tertiary		-0.169***		-0.169***		0.083***		0.083***
		(0.017)		(0.017)		(0.024)		(0.025)
**Country of birth respondent (ref = France)**								
Southern Europe		0.174***		0.453***		0.344***		0.521***
		(0.029)		(0.042)		(0.042)		(0.062)
Rest of Europe		-0.114**		0.001		-0.188**		-0.078
		(0.038)		(0.051)		(0.058)		(0.074)
North Africa		0.548***		0.447***		0.744***		0.435***
		(0.025)		(0.038)		(0.034)		(0.055)
Rest of the world		0.170***		0.189***		0.472***		0.405***
		(0.031)		(0.051)		(0.044)		(0.076)
**Respondent's occupational status (ref = never worked)**								
Farmer		0.111***		0.070*		0.346***		0.274***
		(0.028)		(0.029)		(0.046)		(0.048)
Independent		-0.508***		-0.500***		0.324***		0.316***
		(0.026)		(0.026)		(0.037)		(0.037)

White Collar		-0.791***		-0.790***		0.322***		0.324***
		(0.025)		(0.025)		(0.036)		(0.036)
Blue collar		-0.568***		-0.565***		0.203***		0.207***
		(0.019)		(0.019)		(0.034)		(0.034)
Clerk		-0.366***		-0.361***		0.084*		0.092*
		(0.016)		(0.016)		(0.037)		(0.037)
Manual worker		-0.313***		-0.317***		0.235***		0.237***
		(0.020)		(0.020)		(0.032)		(0.032)
**Mother's occupational status (ref = never worked)**								
Farmer			0.030	-0.014			0.012	0.020
			(0.023)	(0.024)			(0.036)	(0.036)
Independent			-0.055*	-0.022			0.023	0.020
			(0.023)	(0.023)			(0.035)	(0.035)
White Collar			-0.084	0.012			-0.038	-0.072
			(0.056)	(0.056)			(0.079)	(0.079)
Blue collar			-0.034	0.062*			-0.021	-0.051
			(0.025)	(0.025)			(0.036)	(0.036)
Clerk			-0.021	0.011			-0.077***	-0.070**
			(0.015)	(0.015)			(0.022)	(0.022)
Manual worker			0.008	0.006			-0.080**	-0.068*
			(0.018)	(0.018)			(0.027)	(0.027)
Not available			-0.138***	-0.146***			-0.200***	-0.191***
			(0.017)	(0.017)			(0.025)	(0.025)
**Mother's country of birth (ref = France)**								
Southern Europe			0.039	-0.131***			0.125*	-0.073
			(0.034)	(0.037)			(0.051)	(0.055)
Rest of Europe			-0.046	-0.029			-0.103†	-0.091
			(0.039)	(0.042)			(0.056)	(0.059)
North Africa			0.328***	0.100*			0.431***	0.213***
			(0.040)	(0.043)			(0.056)	(0.062)
Rest of the world			0.151**	0.035			0.257***	0.058
			0.039	-0.131***			0.125*	-0.073
**Father's occupational status (ref = never worked)**								
Farmer			-0.725***	-0.633***			-0.841***	-0.809***
			(0.063)	(0.063)			(0.097)	(0.097)
Independent			-0.932***	-0.747***			-0.871***	-0.852***
			(0.062)	(0.062)			(0.095)	(0.095)
White Collar			-0.926***	-0.667***			-0.805***	-0.840***
			(0.063)	(0.063)			(0.096)	(0.097)
Blue collar			-0.994***	-0.772***			-0.936***	-0.921***
			(0.063)	(0.063)			(0.096)	(0.096)
Clerk			-0.960***	-0.787***			-0.955***	-0.925***
			(0.062)	(0.062)			(0.095)	(0.095)
Manual worker			-0.786***	-0.660***			-0.903***	-0.848***
			(0.060)	(0.060)			(0.093)	(0.093)
Not available			-0.896***	-0.768***			-0.985***	-0.945***
			(0.061)	(0.061)			(0.094)	(0.094)
**Father's country of birth (ref = France)**								
Southern Europe			-0.066*	-0.178***			-0.019	-0.126*
			(0.032)	(0.033)			(0.048)	(0.050)
Rest of Europe			-0.100*	-0.113**			-0.056	-0.049
			(0.039)	(0.041)			(0.056)	(0.059)
North Africa			0.185***	0.040			0.274***	0.153**
			(0.039)	(0.041)			(0.055)	(0.058)
Rest of the world			0.002	-0.067			0.138*	-0.004
			(0.046)	(0.050)			(0.069)	(0.074)
**Constant for each number of siblings**								
1 sibling	-1.955***	-2.367***	-2.829***	-3.116***	-1.772***	-1.503***	-2.715***	-2.441***
	(0.027)	(0.030)	(0.066)	(0.067)	(0.039)	(0.049)	(0.100)	(0.104)

2 siblings	-0.758***	-1.161***	-1.629***	-1.908***	-0.783***	-0.508***	-1.722***	-1.443***
	(0.026)	(0.030)	(0.065)	(0.066)	(0.038)	(0.049)	(0.100)	(0.104)
3 siblings	0.739***	0.358***	-0.125	-0.384***	0.749***	1.036***	-0.178	0.106
	(0.026)	(0.029)	(0.065)	(0.066)	(0.038)	(0.049)	(0.100)	(0.104)
4 siblings	1.960***	1.600***	1.103***	0.861***	2.089***	2.387***	1.176***	1.463***
	(0.026)	(0.030)	(0.065)	(0.066)	(0.040)	(0.050)	(0.100)	(0.104)
5 siblings	2.860***	2.512***	2.008***	1.774***	3.147***	3.453***	2.243***	2.532***
	(0.028)	(0.031)	(0.066)	(0.067)	(0.043)	(0.053)	(0.101)	(0.105)
6 siblings	3.577***	3.236***	2.728***	2.500***	4.002***	4.313***	3.105***	3.394***
	(0.030)	(0.033)	(0.067)	(0.068)	(0.049)	(0.058)	(0.103)	(0.108)
7 siblings	4.246***	3.909***	3.400***	3.174***	4.767***	5.080***	3.874***	4.163***
	(0.033)	(0.036)	(0.068)	(0.069)	(0.059)	(0.066)	(0.108)	(0.112)
8 siblings	4.878***	4.543***	4.033***	3.809***	5.547***	5.861***	4.656***	4.945***
	(0.039)	(0.041)	(0.071)	(0.072)	(0.076)	(0.082)	(0.119)	(0.122)
9 siblings	5.529***	5.195***	4.685***	4.461***	6.283***	6.598***	5.393***	5.683***
	(0.048)	(0.050)	(0.076)	(0.077)	(0.103)	(0.107)	(0.137)	(0.140)
10 siblings	6.167***	5.834***	5.324***	5.101***	7.125***	7.441***	6.237***	6.527***
	(0.061)	(0.062)	(0.085)	(0.086)	(0.151)	(0.154)	(0.176)	(0.178)
11 siblings	6.778***	6.445***	5.935***	5.712***	7.840***	8.157***	6.952***	7.242***
	(0.079)	(0.080)	(0.099)	(0.099)	(0.212)	(0.214)	(0.230)	(0.232)
Constant	0.770***	0.819***	0.914***	1.164***	0.740***	0.751***	0.666***	0.966***
	(0.009)	(0.009)	(0.010)	(0.020)	(0.014)	(0.014)	(0.018)	(0.032)
N	136,735	136,735	136,735	136,735	62,176	62,176	62,176	62,176

Note: † p<0.1* p<0.05; ** p<0.01; *** p<0.001. Father's and mother's professional status are not available when the respondent did not know their parent's profession (e.g. if the parent died when they were young, or was not present).

Sample: women aged 45–89 at survey; men aged 45–79 at survey.

Source: Enquête Famille et Logements 2011 (EFL, INED-INSEE)

Interpretation: For a one-unit increase in the number of siblings, the number of children is expected to change by 0.117 (model 4 for women) in the ordered log-odds scale, while holding the other variables in the model constant.

Table A3: Poisson model of completed fertility, by sex: Interaction of birth cohort and total number of siblings (Coefficients, standard error in parenthesis)

	**Women**				**Men**			
	Model 1	Model 2	Model 3	Model 4	Model 1	Model 2	Model 3	Model 4
**Total number of siblings**								
	0.043***	0.039***	0.040***	0.038***	0.031***	0.029***	0.028***	0.027***
	(0.002)	(0.002)	(0.002)	(0.002)	(0.003)	(0.003)	(0.003)	(0.003)
**Birth cohort respondent (ref = 1932–36)**								
1922–1926	0.004	-0.007	0.006	-0.007				
	(0.015)	(0.015)	(0.015)	(0.015)				
1927–1931	0.005	-0.007	0.004	-0.007				
	(0.013)	(0.013)	(0.013)	(0.013)				
1937–1941	-0.060***	-0.037**	-0.058***	-0.036**	-0.067***	-0.070***	-0.070***	-0.071***
	(0.013)	(0.013)	(0.013)	(0.013)	(0.019)	(0.019)	(0.019)	(0.019)
1942–1946	-0.148***	-0.103***	-0.142***	-0.103***	-0.128***	-0.127***	-0.125***	-0.125***
	(0.012)	(0.013)	(0.013)	(0.013)	(0.018)	(0.019)	(0.019)	(0.019)
1947–1951	-0.200***	-0.146***	-0.192***	-0.148***	-0.177***	-0.171***	-0.175***	-0.172***
	(0.012)	(0.012)	(0.012)	(0.012)	(0.017)	(0.017)	(0.017)	(0.017)
1952–1956	-0.210***	-0.150***	-0.203***	-0.155***	-0.189***	-0.179***	-0.183***	-0.178***
	(0.012)	(0.012)	(0.012)	(0.012)	(0.018)	(0.018)	(0.018)	(0.018)
1957–1961	-0.190***	-0.106***	-0.177***	-0.111***	-0.210***	-0.201***	-0.204***	-0.200***
	(0.012)	(0.012)	(0.012)	(0.012)	(0.018)	(0.018)	(0.018)	(0.018)
1962–1967	-0.208***	-0.104***	-0.191***	-0.112***	-0.272***	-0.253***	-0.260***	-0.252***
	(0.012)	(0.012)	(0.012)	(0.012)	(0.018)	(0.018)	(0.018)	(0.018)
**Interaction birth cohort and total number of siblings (ref = 1932–36)**								
Siblings*1922–1926	-0.001	-0.002	-0.001	-0.001				
	(0.003)	(0.003)	(0.003)	(0.003)				
Siblings*1927–1931	0.002	0.002	0.002	0.002				
	(0.003)	(0.003)	(0.003)	(0.003)				
Siblings*1937–1941	-0.000	-0.001	-0.000	-0.002	0.001	-0.000	0.000	-0.000
	(0.003)	(0.003)	(0.003)	(0.003)	(0.004)	(0.004)	(0.004)	(0.004)
Siblings*1942–1946	-0.001	-0.003	-0.001	-0.003	0.003	0.001	0.001	0.001
	(0.003)	(0.003)	(0.003)	(0.003)	(0.004)	(0.004)	(0.004)	(0.004)
Siblings*1947–1951	-0.001	-0.006*	-0.003	-0.006*	0.002	0.001	0.001	0.001
	(0.003)	(0.003)	(0.003)	(0.003)	(0.004)	(0.004)	(0.004)	(0.004)
Siblings*1952–1956	-0.003	-0.009***	-0.005*	-0.010***	-0.000	-0.002	-0.003	-0.002
	(0.003)	(0.003)	(0.003)	(0.003)	(0.004)	(0.004)	(0.004)	(0.004)
Siblings*1957–1961	-0.006*	-0.015***	-0.010***	-0.015***	-0.003	-0.004	-0.005	-0.005
	(0.003)	(0.003)	(0.003)	(0.003)	(0.004)	(0.004)	(0.004)	(0.004)
Siblings*1962–1967	-0.008**	-0.017***	-0.014***	-0.018***	-0.001	-0.004	-0.007	-0.006
	(0.003)	(0.003)	(0.003)	(0.003)	(0.004)	(0.004)	(0.004)	(0.004)
N	136,735	136,735	136,735	136,735	62,176	62,176	62,176	62,176

Note: † *p*<0.1* *p*<0.05; ** *p*<0.01; *** *p*<0.001. Controls for each model like in Table 3.

Sample: women aged 45–89 at survey; men aged 45–79 at survey.

Source: Enquête Famille et Logements 2011 (EFL, INED-INSEE)

Interpretation: In model 4 for women, one additional sibling increases the difference in the logs of expected number of children by 0.038 units, while holding the other variables in the model constant.

Appendix: Family Size of Parents and Children

a. Women

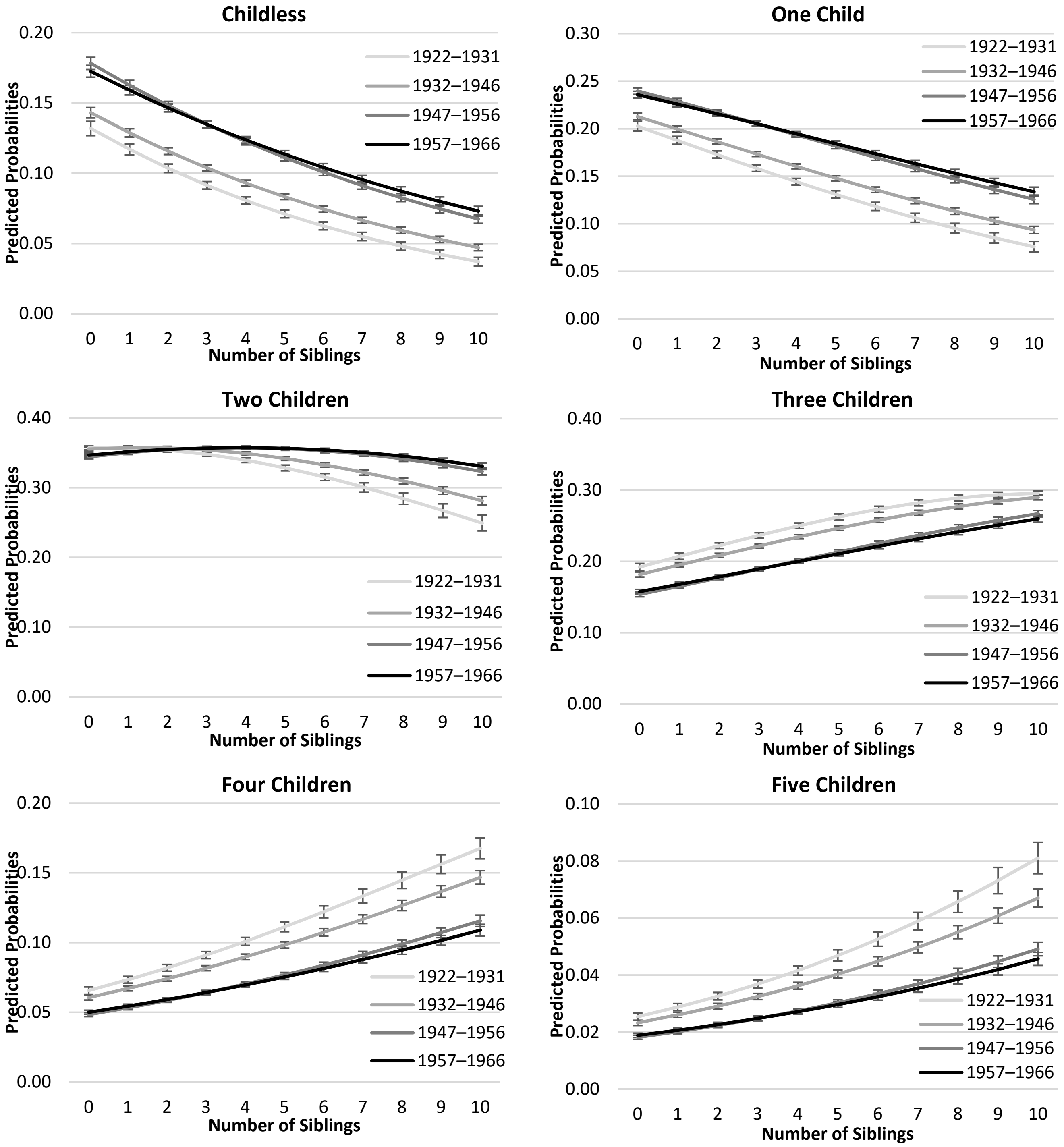

b. Men

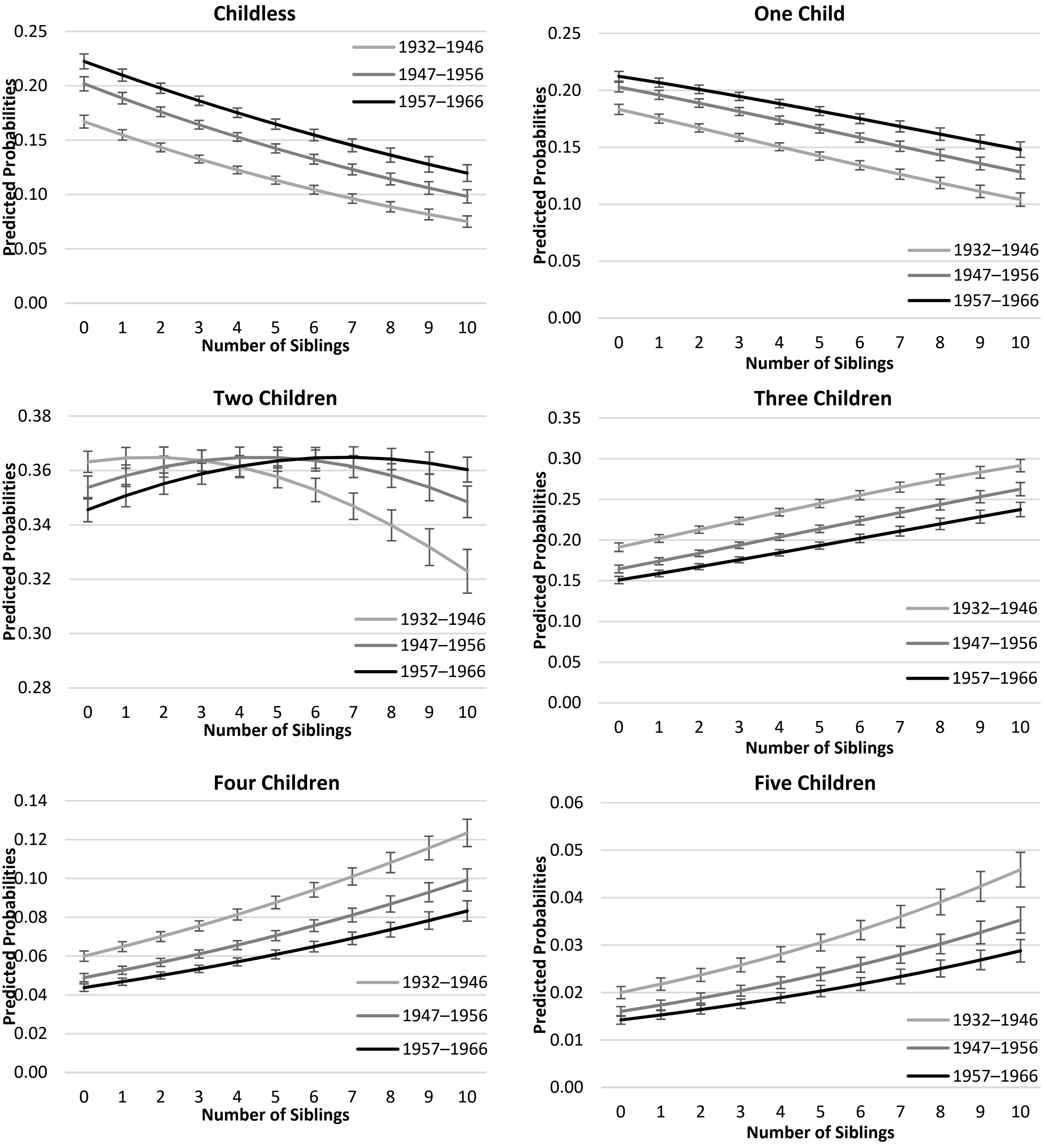

Fig. A1 Predicted probabilities of reaching each family size according to origin family size by birth cohort for women and men (ordered logistic model with controls). The sample is women aged 45-89 and men aged 45-79 at survey. Cohorts are grouped for readability purpose. Additional adjustment variables are respondent's level of education, country of birth, occupational status, mother's and father's country of birth and their occupational status. *Source*: Enquête Famille et Logements 2011 (EFL, INED-INSEE)
